# The calming effect of roasted coffee aroma in patients undergoing dental procedures

**DOI:** 10.1038/s41598-020-80910-0

**Published:** 2021-01-14

**Authors:** Praewpat Pachimsawat, Kanlayanee Tangprasert, Nattinee Jantaratnotai

**Affiliations:** 1 Department of Advanced General Dentistry, Faculty of Dentistry, Mahidol University, Bangkok, 10400 Thailand; 2 Department of Pharmacology, Faculty of Science, Mahidol University, 272 Rama VI Road, Ratchathewi, Bangkok, 10400 Thailand

**Keywords:** Outcomes research, Clinical trial design

## Abstract

Coffee beverage consumption is well-known to exert various health benefits; however, the effects of coffee aroma are rarely explored. This study aimed to investigate the calming effect of inhaling coffee aroma while the patients underwent dental procedures (probing and scaling). Salivary α-amylase (sAA) and cortisol (sCort) levels were measured as proxies of sympathetic nervous system and hypothalamic–pituitary–adrenal axis responses to stress respectively. Blood pressures and pulse rates were recorded. The results showed that undergoing dental procedures could increase sAA and sCort levels of the patients inhaling sham aroma while those inhaling coffee aroma had significantly decreased sAA and sCort levels (40% and 25% differences, respectively). The pulse rates of those inhaling coffee aroma were also lower. Subjective assessment using visual analog scale was in line with objective measures as well. The preference for coffee aroma or the frequency of coffee drinking had no effect on the sAA and sCort responses. This is the first study to provide evidence on the effect of coffee aroma on sAA and sCort levels in patients undergoing dental procedures.

## Introduction

Coffee is one of the most widely consumed beverages in the world and a lot of studies have confirmed its many health benefits both physically^1^ and mentally^2,3^. However, these studies focus on the effects of coffee drinking, not the effect of inhaling coffee aroma. Aromatherapy is a complementary and alternative medicine used to treat various conditions such as stress^4^, anxiety^5^,depression^6^, insomnia^7^, and pain^8^. Even though some people may find the coffee aroma pleasant or can enhance the experience of coffee drinking, there are few studies on the effect of coffee aromatherapy. Coffee essential oil is generally not used in aromatherapy, probably because coffee consumption is well-known to be stimulating and associated with anxiety^9^. However, the major component that induces anxiety is caffeine which is not present in coffee essential oil or in coffee aroma^10^. There is a study using sleep-deprived rats as a stress model that found coffee bean aroma to change the expressions of mRNA and proteins involved in antioxidant and antistress functions suggesting the beneficial effect of coffee aroma in stressful conditions^11^. Another study in mice found the anxiolytic effect of coffee aroma in behavioral tests^12^. However, the anxiolytic effect of coffee aromatherapy has not been explored in humans before.

Dental anxiety and dental fear are the common causes of anxiety and fear in the population worldwide. The terms are often used interchangeably with anxiety indicating anticipated stress and fear indicating actual stress to the situation^13^. In this work, we use the term dental stress to encompass dental stress/anxiety/fear during the whole process of dental care. A number of studies from different countries consistently found high dental anxiety in over 11% of the population with moderate anxiety in about 25% of the population^14–19^. The impact of this could be great as people with high dental anxiety were associated with avoidance of dental visiting, infrequent check-ups, poorer oral health, difficult to manage dental problems, unfavorable attitudes toward dentists, stress in the dentists, and dissatisfaction with one's mouth^13,20^.

Salivary α-amylase (sAA) and cortisol (sCort) can be used as objective measures of stress^21^. It is suggested that sAA be used as a surrogate for the activity of the sympathetic nervous system (SNS) because it is produced more when the SNS is stimulated^22^. While cortisol is the end product of the hypothalamus–pituitary–adrenal (HPA) axis so its level is directly correlated with the activity of HPA axis^23^. Both sAA and sCort have been shown to increase in various stress models and situations^24,25^ as well as in dental stress^26^. Previous studies have revealed the beneficial effect of aromatherapy in reducing dental stress^27–29^. Aromatherapy could be an inexpensive, noninvasive, and convenient measure to help relieve dental stress which might increase compliance to dental care that finally lead to improved oral health in patients. In the current study, we aimed to compare the effect of coffee aromatherapy or sham aroma on sAA and sCort levels as well as physiological measures during dental procedures that are known to induce stress.

## Results

### Sample characteristics

The patients in the control group had similar baseline characteristics compared with the coffee group as summarized in Table 1 (n = 71). The patients’ ages ranged from 19–65 years old. They had BMI between 17.82 and 40.79 kg/m^2^. From modified dental anxiety scale (MDAS), none of the patients had dental phobia (MDAS > 19) and four had high dental anxiety (MDAS > 14). The range of MDAS was between 5 and 17. About half of the patients were regular coffee drinkers, i.e. they drank coffee at least once a week (n = 36). Among the patients who drank at least 5 times per week (n = 25), 88% liked the coffee aroma while only 39.13% of the patients who drank less frequently or did not drink (n = 46) found the coffee aroma pleasant. Only six patients did not like coffee aroma, among these, four did not drink coffee and two drank occasionally. All patients received no harm or unintended effects from the experiment.

**Table 1:** Baseline characteristics of the subjects (mean ± SEM).

Parameters	All (n = 71)	Control (n = 36)	Coffee (n = 35)
Age (years)	36.21 ± 1.51	36.25 ± 2.08	36.17 ± 2.22
Sex: female	56 (78.9%)	28 (77.78%)	28 (80%)
BMI (kg/m^2^)	24.36 ± 0.57	24.88 ± 0.86	23.82 ± 0.75
Systolic blood pressure (mmHg)	120.56 ± 1.81	122.53 ± 2.68	118.54 ± 2.42
Diastolic blood pressure (mmHg)	70.96 ± 1.15	72 ± 1.68	69.89 ± 1.56
Pulse rate (beats/min)	76.38 ± 1.15	76.28 ± 1.67	76.49 ± 1.60
MDAS	9.41 ± 0.38	9.53 ± 0.55	9.29 ± 0.52
**Coffee drinking habit**			
Occasional or don’t drink	35 (49.30%)	18 (50%)	17 (48.57%)
1–4 times/week	11 (15.49%)	7 (19.44%)	4 (11.43%)
≥ 5 times/week	25 (35.21%)	11 (30.56%)	14 (40%)
**Coffee aroma**			
Like	40 (56.34%)	15 (41.67%)	25 (71.43%)
Dislike	6 (8.45%)	5 (13.89%)	1 (2.86%)
Neutral	25 (32.21%)	16 (44.44%)	9 (25.71%)

### Levels of sAA after full mouth probing

**Figure 1:**
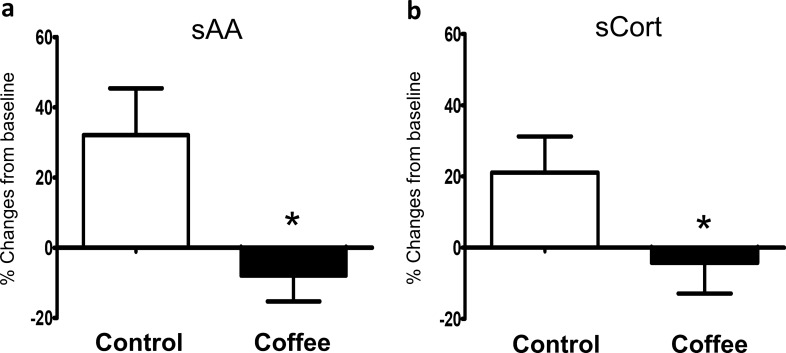
The relative differences (%) from baseline of (**a**) salivary α-amylase (sAA) and (**b**) salivary cortisol (sCort) in control and coffee inhaling groups. *p* < 0.05.

The patients collected saliva before and after full mouth probing. The levels of sAA were measured on-site with a hand-held biosensor. Since the baseline sAA varied over a wide range (5–150 u/mL), the relative changes from baseline were used. As expected after a stressful event, patients in the control group had increased sAA levels after probing (32.08 ± 13.34%); however, patients in the coffee group demonstrated decreased sAA levels after probing (− 7.92 ± 7.35%). The relative differences in sAA levels before and after probing were significantly different between control and coffee groups (*p* = 0.002) as shown in Fig. 1a.

### Levels of sCort after full mouth probing and scaling

The patients collected whole mouth saliva before and after all the dental procedures (full mouth probing and scaling) for sCort measurement. Note that sAA levels were measured after probing only. We analysed sCort levels after sAA measurement as sCort was shown to demonstrate a delayed response compared with sAA^30^. Baseline sCort levels were in the range of 0.045–0.581 µg/dL. Similar to changes in sAA levels, sCort levels increased in the control group after the dental procedures (21.09 ± 10.16%), while sCort levels in the coffee group decreased (− 4.27 ± 8.64%). The relative changes in sCort before and after all dental procedures between control and coffee groups were significantly different (*p* = 0.02) as shown in Fig. 1b.

### Physiological parameters after dental procedures

**Figure 2:**
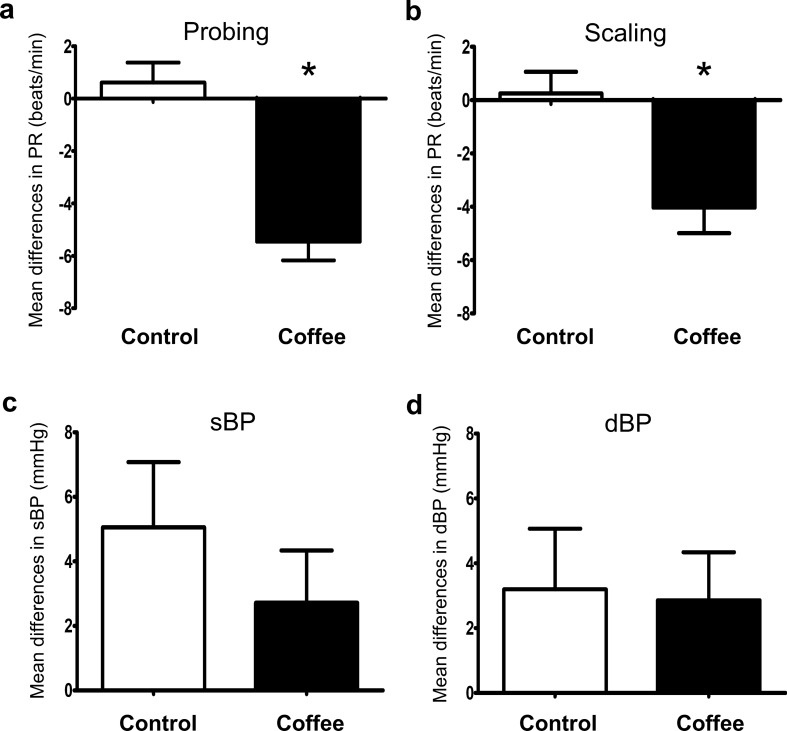
The mean differences in pulse rate after probing (**a**), scaling (**b**) and in systolic (**c**) and diastolic blood pressure (**d**) after probing and scaling in control and coffee inhaling groups. *p* < 0.05.

The patients’ pulses were measured 3 times: before dental procedures, after probing, and after scaling. While the blood pressures were measured 2 times, i.e. before and after all dental procedures. Figure 2 showed the differences in pulses and blood pressures of the control and the coffee groups. In the control group, the pulse rates did not change after either probing (*p* = 0.482) or scaling (*p* = 0.760), while in the coffee group, the pulse rates significantly decreased both after probing (*p* < 0.001) and scaling (*p* = 0.018). The differences in pulse rate changes before and after both probing and scaling were significant between control and coffee groups (*p* < 0.001). On the other hand, the blood pressures were not significantly different between control and coffee groups (*p* = 0.371 for systolic blood pressure, and *p* = 0.888 for diastolic blood pressure).

### Subjective assessment

**Figure 3:**
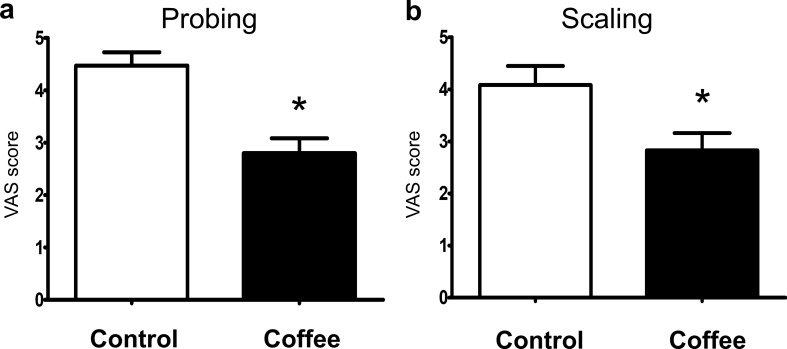
The mean scores of VAS after probing (**a**) and scaling (**b**) in control and coffee groups. *p* < 0.05.

After each dental procedure, the patients rated their current feeling of distress on a visual analog scale (VAS) with 10 indicating greatest distress and 0 indicating no distress. Of all 71 patients, only two and seven patients expressed marked distress (VAS score ≥ 7) after probing and scaling, respectively. As shown in Fig. 3, the patients in the coffee group showed significantly lower levels of distress compared to the control group. The patients’ scores on VAS after probing vs VAS after scaling were significantly correlated (Spearman’s rho = 0.417, *p* < 0.000).

### Coffee habits

The frequency of coffee drinking or the preference to coffee aroma had no association with the levels of sAA and sCort. Subgroup analyses revealed that patients who seldom drank coffee or who did not find coffee aroma pleasurable still benefited from inhaling coffee aroma during dental procedures as the levels of sAA and sCort were similar to those who regularly drank coffee or who liked coffee (Supplementary Table 1).

## Discussion

People inhaling coffee aroma while undergoing dental procedures had decreased sAA and sCort levels compared with people inhaling sham aroma which showed increased sAA and sCort levels, accounting for 40% and 25% differences, respectively. The pulse rates decreased in the patients smelling coffee aroma compared with control as well. The subjective measurement of stress (VAS) was also different between control vs coffee groups with the coffee group demonstrating less stress while undergoing dental procedures. This is the first study to examine the effect of coffee aroma on sAA and sCort levels using dental treatment as a stress model.

Our results show that coffee aromatherapy not only ameliorated enhanced stress reactivity but actually reversed it as shown with the changes in sAA and sCort levels. Lower sAA and sCort levels indicated less SNS and HPA axis reactivity which was found to be associated with many disease conditions^31^. In a previous randomized controlled crossover trial, it was shown that children undergoing dental treatment exhibited increased sCort levels and pulse rates, while administering orange aromatherapy resulted in no changes in sCort and pulse rates after dental procedures^27^. We also found the pulse rates to decrease in a similar manner with sAA and sCort levels. Subjective assessment of stress was also in compliance with sAA and sCort levels further confirming the beneficial effect of coffee aromatherapy. Our results are in line with other odors used for dental care; orange or lavender aromatherapy has been shown to improve the mood of the patients while waiting in the dental office as assessed from the self-reported questionnaires^28,29^. A recent study using coffee aroma also found that it can enhance cognitive performance in healthy volunteers^32^. To our knowledge, there is no other study on the effect of coffee aromatherapy.

From MDAS results, no patients in the current experiment had dental phobia and only 5.63% had high dental anxiety. These numbers were lower than those found in the general population in other studies probably because these patients would avoid dental treatment in the first place. Our results found that MDAS was not related to the levels of sAA and sCort or any other parameters investigated probably because most patients did not have dental anxiety. Previous work reported various results regarding the correlations of dental anxiety and sAA or sCort levels. A previous study did not find significant correlations between Dental Anxiety Scale and sAA or sCort levels but the assessment was not performed before or during dental procedures^33^. Another study measured sCort levels before performing emergency dental care and found that sCort levels were not correlated with MDAS but with pain before dental treatment^34^.

Our results support the use of an on-site portable sAA biosensor to evaluate dental stress. A previous study on dental surgery-induced stress also employed this device to measure the levels of sAA^35^. The current sAA results are consistent with the previous studies of different stress models such as driver’s fatigue, maternal stress, mental stress, and military training^36–39^. The range of sAA was within the linear reading capacity of the device and comparable with other studies^36,40^. For sCort levels, they were consistently found to be increased before or after various dental procedures^41–43^. Such levels were correlated with the pain associated with each procedure, for example, dental extraction demonstrated the highest sCort levels compared with examination or prophylaxis treatments^43^.

Preferences to coffee aroma or the frequency of coffee drinking had no effect on whether a person will benefit from coffee aromatherapy or not. As our results have proved that the levels of sAA and sCort similarly decreased among patients who did not find coffee aroma pleasurable or who did not regularly drink coffee. This suggests that the effect of coffee aroma was objective and likely dependent on the active volatile compounds in the coffee aroma. The active ingredients in coffee aroma that elicited the calming effect in the current study are not known. There are more than 900 volatile compounds present in roasted coffee^44^ but there is no study exploring the effect of these compounds yet. Also, the constituents in coffee could vary greatly depending on species, geographical origin, cultivation, roasting and preparation process, etc^44^. For coffee as a beverage, there are many studies investigating the beneficial effects of the compounds present in the drink. Among the most studied compounds include chlorogenic acid, caffeine, cafestol, kahweol, and trigonelline which have been found to exert anti-inflammatory and antioxidant properties^45^. However, the volatile compounds inhaled from coffee aroma could have different profiles from what are present in coffee bean oil and coffee beverages.

There are several limitations of our study. Firstly, the researchers were not blinded to the treatment group and even though the patients were not told of the treatment received, they likely could tell by the smell. This could confound the results or the expectation of the treatment received. Secondly, we did not measure both sAA and sCort levels at the same time, making it difficult to compare the time course of these parameters. Also, we were not able to identify the active components in coffee aroma responsible for the observed calming effect. In summary, using coffee aromatherapy appears to be a convenient and effective means to help reduce dental stress. This method can be conveniently implemented on its own or in conjunction with other methods such as music or relaxation techniques. There should be more studies on the roles and the active compounds of coffee aroma.

## Material and Methods

### Subjects

The patients were healthy volunteers who were willing to undergo full mouth probing and scaling. They were recruited from an announcement in the Faculty of Dentistry, Mahidol University. The inclusion criteria included being older than 18 years old without underlying diseases and not taking medication, not pregnant, have at least 20 teeth to undergo probing and scaling, and have a normal sense of smell. The exclusion and withdrawal criteria included allergy to coffee aroma, alcohol consumption during the past 12 h, food or drink other than plain water during the past hour, smoking, and unwillingness to participate in the project at any moment. A total of 71 patients were randomized and completed the experiment between May and October 2017.

### Procedures

**Figure 4:**
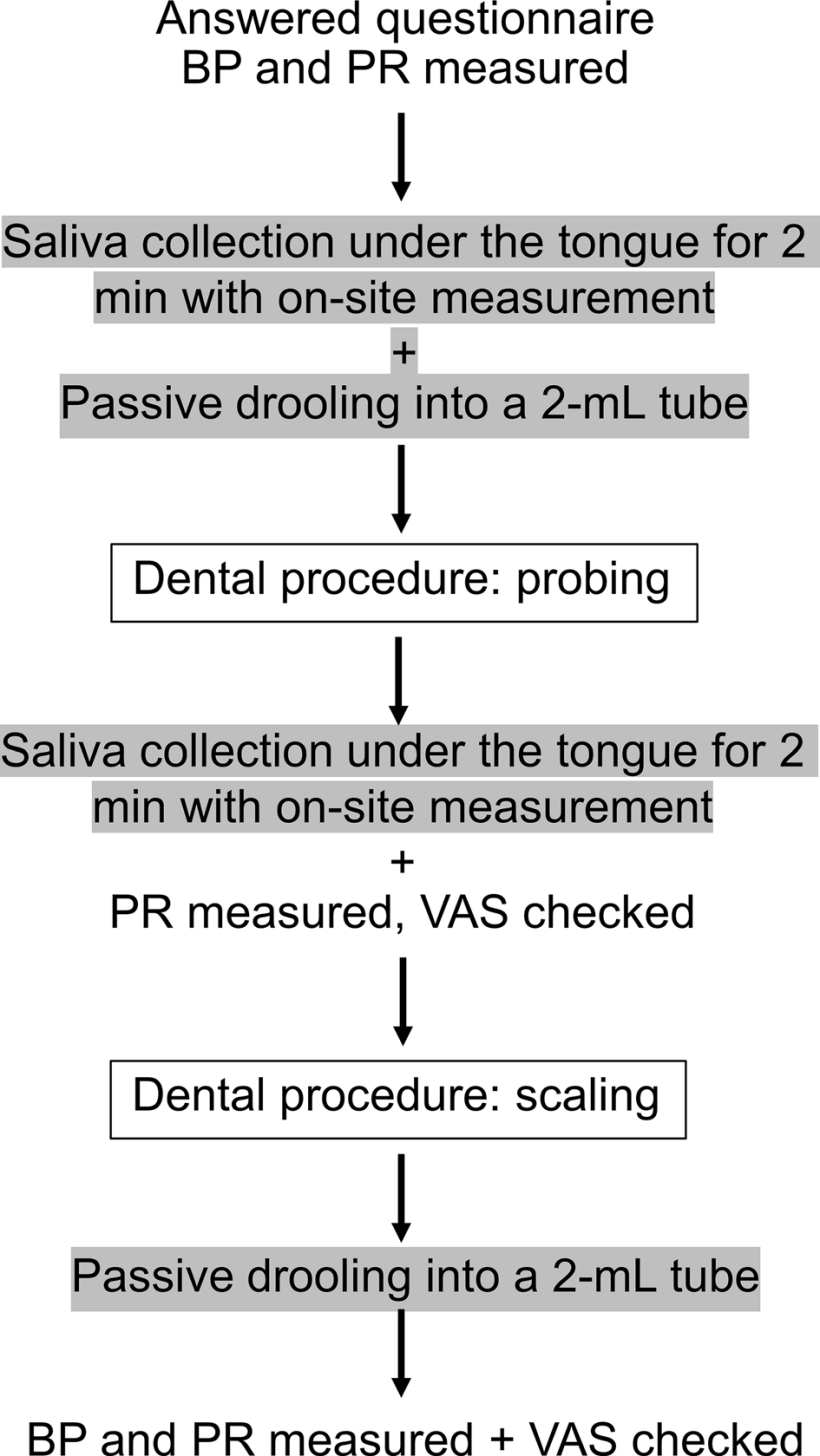
A diagram detailing the protocol. BP, blood pressure; PR, pulse rate; VAS, visual analog scale.

The researcher (KT) employed simple randomization to assign the patients to either coffee aroma or sham aroma group while undergoing dental procedures of full mouth probing and scaling which were expected to induce pain and stress. Periodontal probing assesses periodontal conditions while scaling removes plaque and calculus. Both procedures are routinely performed in dental care. The patients answered a general questionnaire about themselves, the preference and habit of coffee drinking, as well as MDAS (Modified Dental Anxiety Scale). Then the blood pressure and pulse rate were measured. Saliva was collected by under-the-tongue method for immediate measurement and by passive drooling for later measurement in a laboratory. Then the patient was brought into an operating room for the dental procedures. In this room, a diffuser was placed at 60 cm away from the patient’s head and the diffuser was turned on until the end of all the dental procedures which took about an hour. Probing was performed first, then scaling was performed. After each dental procedure, the patients rated their feeling on a visual analog scale (VAS) and saliva was collected again. The experiment was always performed in the afternoon between 1 and 4 PM to reduce the effect of diurnal variation of sAA and sCort levels. The summary of the protocol is shown in Fig. 4. This study was approved by Faculty of Dentistry/Faculty of Pharmacy, Mahidol University, Institutional Review Board (MU-DT/PY-IRB 2017/020.2103). The trial was registered in clinicaltrials.in.th, registration number TCTR20170523003 on 21 May 2017. The study was conducted according to the principles of the Declaration of Helsinki. All patients gave written informed consent.

### Coffee aroma distribution

Roasted coffee beans essential oil was purchased from Eden Botanicals, Petaluma, CA, USA. Its source was from organic *Coffea arabica* beans grown in Italy and was extracted with CO_2_ method. A commercially available aroma diffuser (Muji, Japan) was used to diffuse the coffee aroma at the dilution of 1:150 with distilled water. The diffuser was turned on during a whole dental procedure. The size of the operating room was 16 m^2^. In a control group, no coffee essential oil was added but the diffuser was still turned on. The patients were blinded to the treatment allocation.

### Saliva collection and analysis

Each patient provided four samples of saliva by two methods. The first one was to hold the collector strip under the tongue for 2 min. Then this collector strip was used to measure sAA levels on-site right away with a handheld biosensor as explained in a previous study^46^. Briefly, the biosensor is a miniaturized optical reading machine that employs an enzyme kinetic method to measure amylase activity. The reaction product is measured at 430 nm. The biosensor can measure sAA levels correctly in the linear range between 0 and 200 U/mL with 10.2% coefficient of variation^47^. The second method was to allow the patients to pool the saliva for 4 min and passively drool the saliva into a 2-mL tube. This sample was later kept at -80 °C until further analysis. The frozen saliva was thawed and centrifuged at 1500 g for 15 min prior to measurement. The supernatant was retrieved for measuring sCort levels using a competitive enzyme immunoassay kit (Salimetrics, State College, PA, USA). The procedure was performed according to the manufacturer’s instruction. The amount of cortisol enzyme conjugate which is inversely proportional to the amount of cortisol present is read at 450 nm with a microplate reader (Varioskan Flash Multimode Reader, Thermo Fisher Scientific, Rockford, IL, USA) and computed using a four-parameter curve fit.

### Subjective assessment

Modified Dental Anxiety Scale (MDAS)^48^ is a generally used tool to stratify patients’ anxiety. It consists of 5 questions for patients to rate if common dental procedures are about to be performed. The scale ranges from not anxious to extremely anxious with the score ranges from 5 to 25. The score of ≥ 14 indicates high dental anxiety and the score of ≥ 19 indicates dental phobia^14^.

Visual Analog Scale (VAS) is a linear scale with a ruler ranging from 1 to 10 to determine the intensity of distress after each dental procedure. The score of 0 indicates well-being without any distress. The score of 10 indicates extreme distress. The patients assessed their distress on the VAS before and after completion of all dental procedures (full mouth probing and scaling).

### Statistical analysis

The results were presented as mean ± SEM. Kolmogorov–Smirnov test was used to determine the normality of the samples. Independent t-test or Mann–Whitney test was used to compare the mean differences depending on the normality distribution of the data. Correlations were assessed using Spearman’s nonparametric analysis. Statistical analysis was performed using SPSS statistics program version 21 (IBM, Armonk, NY, USA). The significant level was considered at *p* value < 0.05.

## Supplementary Information

Supplementary Information.

## Data Availability

The datasets generated during and/or analysed during the current study are available from the corresponding authors on reasonable request.
