# Supplementary material for: The calming effect of roasted coffee aroma in patients undergoing dental procedures
Source: Sci Rep. 2021 Jan 14;11:1384. doi: 10.1038/s41598-020-80910-0 (PMC7809118; doi:10.1038/s41598-020-80910-0)
Supplement: Supplementary file 1 — Supplementary Information. [file 41598_2020_80910_MOESM1_ESM.docx]

**The calming effect of roasted coffee aroma in patients undergoing dental procedures.**

Praewpat Pachimsawat, Kanlayanee Tangprasert, Nattinee Jantaratnotai

**Supplementary table 1. Subgroup analyses of relative sAA and sCort levels (% from baseline).**

For participants who did not drink or seldom drank coffee

Control (n = 18) Coffee (n = 17) p

sAA 39.62 ± 19.62 -15.16 ± 5.79 .000

sCort 9.34 ± 9.72 -15.93 ± 5.59 .080

For participants who did not find coffee aroma pleasurable

Control (n = 21) Coffee (n = 10) p

sAA 27.13 ± 18.04 -22.49 ± 15.24 .004

sCort 29.98 ± 14.10 -22.72 ± 6.34 .003
